# Wireless Powered Surface Acoustic Wave Platform for Achieving Integrated Functions of Fogging/Icing Protection and Monitoring

**DOI:** 10.1021/acsami.4c14669

**Published:** 2024-10-25

**Authors:** Huiling Ong, Feixuan Yang, Luke Haworth, Chi Zhang, Jikai Zhang, Haimeng Wu, Jikui Luo, Qiang Wu, Yongqing Fu

**Affiliations:** † Faculty of Engineering and Environment, Northumbria University, Newcastle upon Tyne NE1 8ST, U.K.; ‡ College of Information Science and Electronic Engineering, Zhejiang University, Hangzhou 310027, China

**Keywords:** surface acoustic waves, frequency shift, SAW sensing, ice monitoring, rime ice

## Abstract

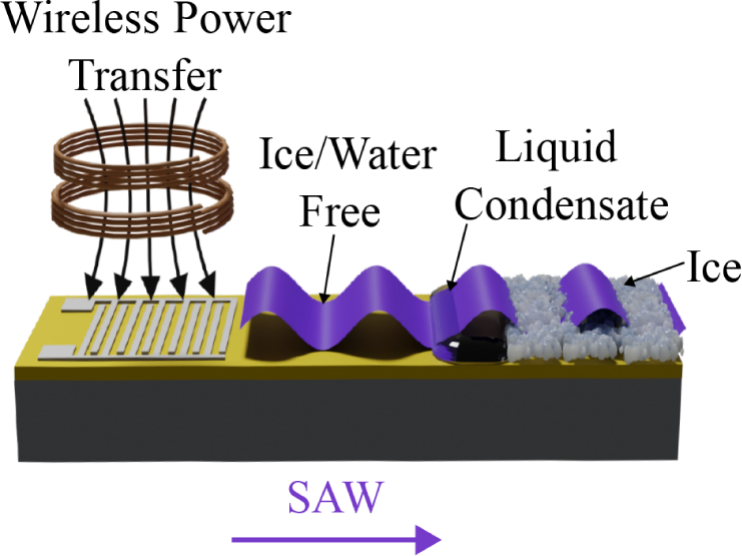

In this study, we introduced an integrated approach using piezoelectric thin film-based surface acoustic wave (SAW) and wireless power transfer (WPT) technologies, designed for both passive monitoring and active defogging/icing functions. We systematically investigated the resonant frequency shifts of ZnO/glass SAW devices, establishing their correlations with variations in humidity and temperature under cold conditions. Acoustic waves generated through the ZnO/glass SAW device were used for defogging and deicing functions with effects of RF powers and acousto-heating thoroughly evaluated. More significantly, the WPT system was successfully applied for achieving defogging and deicing functions, with its performance comparable to that of conventional wired SAW systems. Our findings demonstrated that the WPT SAW system significantly minimizes localized acousto-heating effects, although the time taken for both defogging and deicing was slightly longer than the wired system. This work represents a significant advancement in developing multifunctional, optically compatible, and wireless-integrated solutions for SAW based ice protection.

## 1 Introduction

Fogging, condensation, icing, and frosting on glass surfaces cause poor visibility and hazard issues in lenses, windscreens, aeroplane windows, and solar panels.^1−5^ Water condensation, subsequent icing, and frosting easily occur on cold structural surfaces with surface temperatures at or below the dew point in situations of high relative humidity (RH) in a cold condition.^6−8^ They often create safety hazards due to substantially reduced vision and endanger people’s health and safety.^9,10^ Increasing extreme weather conditions caused by climate change result in significant accretion of ice and snow on many surfaces (i.e., automobile, train, and aeroplane windows and windscreens, camera lenses, and traffic lights), resulting in poor operability and safety hazards.^1,4^ To solve these problems, various active and passive methods have been applied to the glass components. Active systems include external energy sources or heating, dynamic perturbation methods, or mechanical methods, whereas passive systems include superhydrophobic and icephobic surfaces, micro- and nanostructures, or chemicals or refrigerants.^9−13^ However, these methods often tend to consume a huge amount of energy or pose pollution or corrosion issues.^8,9,12,14−16^ Some commonly investigated superhydrophobic coatings or surfaces have shown poor mechanical/adhesion properties and durability issues.^17−19^

Precise sensing and monitoring fogging, frosting, and ice formation are extremely challenging due to various environmental influences, including temperature, humidity, and pressure, which can affect the phase transitions of water molecules among different states/conditions, e.g., fog, moisture, snow, ice, and frost.^20^ There have been several methods developed to study moisture/ice monitoring and formation of ice/frost, most of which were based on *in situ* measurements of changes in parameters of the proposed sensor due to mass loading, dielectric constants, inductance, conductance, optical properties, and even vibration frequencies.^20^

**Figure 1:**
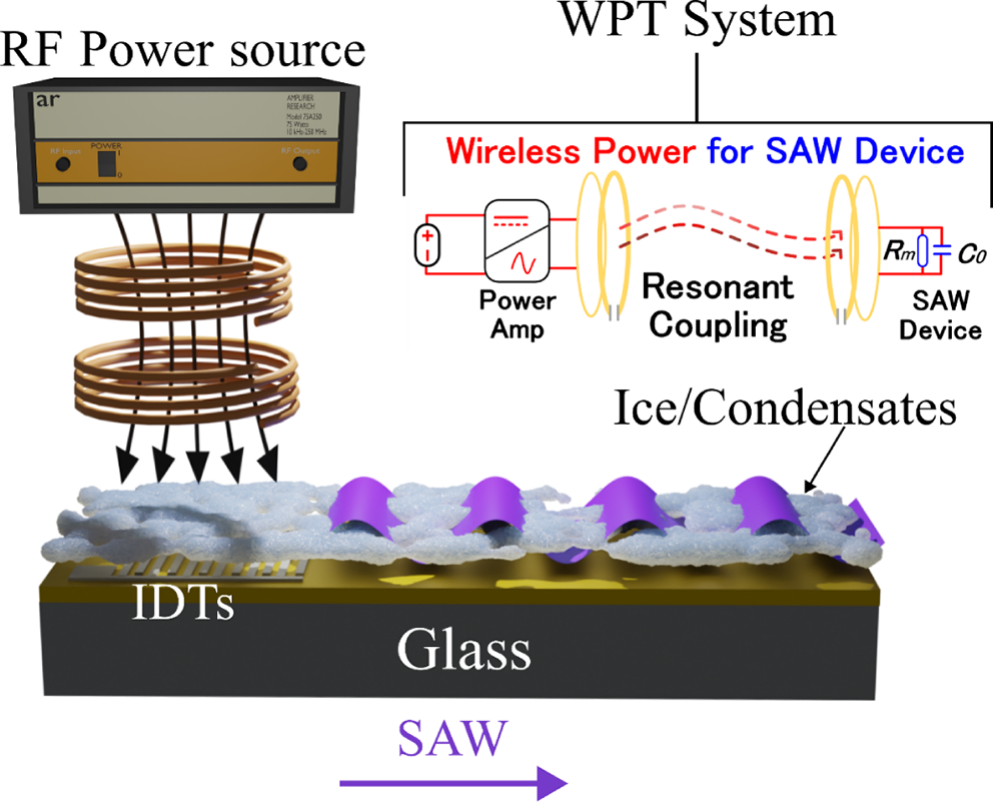
Illustration of monitoring and deicing principles for SAW and WPT systems with the embedded compensation equivalent circuit.

Recently, integration of thin film-based surface acoustic wave (SAW) devices on structural surfaces, such as glass has been reported to achieve antifogging, anti-icing, and deicing functions, as well as sensing functions.^21−24^ For example, in fogging mitigation, SAW agitations induce internal streaming of water droplets, which can effectively remove condensation or moisture from the surface.^24^ The localized acousto-thermal effect can be utilized to delay or stop the formation of frost, fogging, and ice. As for deicing (Figure 1), mechanical vibrations from the SAW acoustic energy can cause interfacial separation or crack formation.^23−25^ Due to significant vibrations at the interfaces, interfacial localized acousto-heating can also be induced leading to shrinkage or removal of ice.^23−25^ At the same time, the changes of condensation, formation or removal of fogging, and icing can all be *in situ* monitored using the frequency shifts of the SAW devices (Figure 1).^20^

Although thin film SAW devices can realize most of the aforementioned functions, there is a critical issue that it is difficult to use cables and wires to connect SAW devices that are integrated into lenses, windows, or glass-based PV panels. The requirements necessitate the miniaturization and portability of SAW devices, thus making integrating wireless power transfer (WPT) technology a crucial function. WPT technology has recently been developed for applications in many fields, including electronics, medicine, automobile, communication, sensing, and security, and it can also be integrated with the SAW technology.^26−31^ WPT uses an electromagnetic near field in a short-range region with the advantage of a high transmission efficiency. There have been initial studies that integrate the WPT with SAWs. For instance, Biryukov et al. utilized a WPT system to enhance the rotation effect of the piezoelectric tube as a function of SAW propagation loss per period, which can be varied by several orders of magnitude.^32^ Tan et al. developed wireless sensing method via a passive wireless triboelectric sensor, which consisted of a triboelectric nanogenerator, an RF remote reader, and a SAW resonator.^33^ However, WPT-based thin film SAW devices have seldom been optimized and applied for icing protection applications.

In this study, a WPT system was meticulously designed to match the resonant frequency of the SAW device, ensuring optimal performance at its operating frequency, as shown in Figure 1. The WPT system incorporates a resonant circuit precisely tuned to align with the SAW device’s resonant frequency. The power amplification unit modulates and amplifies the power in the transmitter, which was wirelessly transferred to the SAW device via the receiver circuit. We then developed an integrated thin film SAW and WPT system on glass substrates for defogging, deicing, and monitoring applications. For sensing purposes, we investigated the frequency shifts caused by the combination of temperature, electrical, and mass loading effects under various cold conditions. We then focused on demonstrating deicing and defogging functions with ZnO/glass SAW devices and evaluated the wireless-powered capabilities.

## 2 Experimental Details

### 2.1 Preparation and Characterization of SAW Devices on Glass

ZnO thin films of ∼4.5 μm thick was deposited on four in. borosilicate glass wafers via a direct current (DC) magnetron sputter (NS3750, Nordiko) with a zinc target (99.99% purity). Before the film deposition, the glass substrates were cleaned with acetone, isopropanol (IPA), and deionized (DI) water and finally dried using nitrogen gas. The distance between the glass substrates and the ZnO target was 20 cm. The vacuum pressure of the sputter chamber was maintained at ∼0.35 Pa. During the deposition process, the surface of the Zn target was oxidized and sputtered with ZnO film with an Ar/O_2_ flow ratio of 10/15 SCCM (standard cubic centimeter per minute). The plasma power was set at 400 W. More details of the film deposition and characterizations can be found in ref.^24,34,35^ Cross-sectional morphology of ZnO films was examined using a scanning electron microscope (SEM, Tescan Mira 3). An X-ray diffractometer (XRD, Rigaku) was used to examine its crystalline structure, utilizing a Ni-filtered Cu Kα radiation X-ray source (λ = 1.5406 Å) operated at 40 kV and 30 mA. A UV–vis spectrophotometer (NKD-8000, Aquila) was used to characterize the optical characteristics of the ZnO film-deposited glass, and Raman spectrometer (Thermo Scientific DXR3) was used to obtain the Raman spectra with a laser beam wavelength of 532 nm.

To fabricate SAW devices, the interdigital transducers (IDTs) were patterned on top of a ZnO/glass substrate via conventional photolithography and lift-off processes. A bilayer of Cr/Au with thicknesses of 20/100 nm was prepared as the electrode via a thermal evaporator (Edwards AUTO306). The designed IDTs had 30 pairs of fingers with three different wavelengths (10, 16, and 100 μm). The reflection spectra (*S*_11_) of the ZnO/glass SAW devices were measured using a network analyzer (Keysight, FieldFox N9913A). The temperature coefficient of frequency (TCF) of the ZnO/glass SAW devices was obtained by using the following equation:


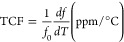
1

The TCF of the ZnO/glass SAW devices was measured by monitoring their resonant frequencies as the temperature was changed from 60 to 20 °C in a controlled environment using a laboratory oven (Carbolite^TM^ LHT).

### 2.2 Icing/Fogging Tests Using Cabled SAW Device

ZnO/glass SAW devices (SAW in short hereafter) were used for defogging experiments and were placed in the middle of a bespoke cold chamber. To produce condensation on the surface of the SAW device, a nebulizer (Omron ultrasonic nebulizer NE-U17) was employed with a measured relative humidity (RH) of 100%. Humid air was continually introduced into the cold chamber for a given period (e.g., one min) through a tube. Condensation was seen to form on the surface of the SAW device after exposure to humid air. The SAW device was exposed to various RF powers (0.63 to 3.41 W) to eliminate moisture, while the duration required for the fog to be removed was recorded. For simplicity, the SAW devices were electrically connected via wires for this experiment.

For the deicing of rime ice on glass substrates, the experiments were conducted in the same bespoke cold chamber maintained at a stable icing environment. The SAW device was placed on a cold plate in the chamber, set at a temperature of −6.5 ± 0.3 °C. Different relative humidity (RH) levels were achieved using the nebulizer that generated water aerosols. Before the start of icing, the SAW device was cooled and maintained at −6.5 ± 0.3 °C for at least 30 min in advance. The moisture from the icing process was measured with an RH value of 90%. The deicing tests using the rime ice were performed with different SAW powers (0.63 to 4.01 W) applied to the SAW device. After the formation of rime ice on the surface of the SAW device, a signal generator (Aim TTi, TG5011A) was used to generate an RF signal at the SAW device’s resonant frequency, which was amplified using a power amplifier (Amplifier Research, Model 75A250). An RF power meter (Racal Instruments 9104) was used to measure the RF power applied to the IDTs of the SAW devices. A video camera (IDS UI-3270LE-C-HQ) with a Navitar 12x objective lens was used to record the changes in ice morphology from the top and side views of the glass device.

To monitor the frequency shifts of SAW devices when subjected to changes in icing conditions, including temperature and humidity, the bespoke cold chamber was used. The printed circuit board (PCB) with the SAW device (resonant frequencies of 28.19, 162.58, and 244.21 MHz) was directly positioned inside the cold chamber. The network analyzer was placed outside of the cold chamber and used to record the frequency readings continuously. A thermocouple was placed inside the cold chamber and attached to the surface of the SAW device for temperature measurement during the operation. The following four different sensing and monitoring studies were performed.

In case 1, the temperature effects on the SAW devices without any humidity effects were studied. The cold chamber was cooled from room temperature (∼23.0 ± 0.3 °C) to low temperature (∼−9.0 ± 0.3 °C), with nitrogen gas flowing in the chamber at a steady flow rate of 1.0 m^3^/min. The nitrogen gas allows for the humidity to be reduced in the chamber to create a dryer atmosphere as it displaces the more humid air. Although nitrogen gas was used, it could not eliminate all moisture and condensation from occurring, resulting in an RH ∼ 5%. In this case, both the frequency and temperature were recorded simultaneously.

In case 2, the humidity effects were studied without significant temperature influences (i.e., without cooling the cold chamber). The humid air to generate different relative humidities (RH 60, 90, and 100%) was blown into the cold chamber via the nebulizer at room temperature (∼23.0 ± 0.3 °C) for 20 min. In this case, only the frequency was recorded as the temperature was stable.

In case 3, the effects of both the humidity and temperature were explored in a sequence test. The SAW device was placed on a cold stage in the chamber. The cold chamber was first cooled from (∼23.0 ± 0.3 °C) to a low temperature (∼−9.0 ± 0.3 °C). Then, the humid air with different relative humidity levels (RH 60, 90, and 100%) was introduced into the cold chamber via the nebulizer for 20 min. In this case, both the frequency and temperature were recorded simultaneously.

In case 4, the synergy of humidity and temperature effects on the SAW devices was evaluated concurrently. The cold chamber was cooled simultaneously with humid air exposed to different relative humidities (RH 60, 90, and 100%) via the nebulizer for 20 min, starting from room temperature (∼23.0 ± 0.3 °C) to a low temperature (∼ −9.0 ± 0.3 °C). In this case, both the frequency and temperature were recorded simultaneously.

### 2.3 Wireless Power Transfer System

**Figure 2:**
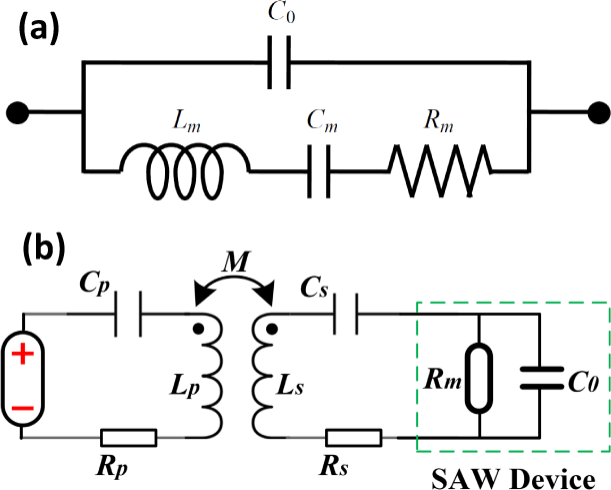
(a) Electrical equivalent circuit of a one port SAW resonator; (b) equivalent circuit of SAW device in resonant state.

In this work, an electrical equivalent circuit model has been used to describe the resonant behavior of a SAW resonator. Figure 2a shows the corresponding equivalent circuit at the resonance. Near the resonant frequency, the SAW device behaves as a network consisted of a static capacitance *C*_0_, a moving inductance *L*_m_, a moving capacitance *C*_m_, and a moving resistance *R*_m_ (where *C*_0_ is the electrostatic capacity representing the fixed dielectric capacity of the resonator, *L*_m_ and *C*_m_, respectively, correspond to the contributions of inertial and elastic forces, and *R*_m_ is the moving resistance corresponding to the contribution of damping force).^36,37^ These parameters can be calculated from the resonance frequency *f*_r_, antiresonance frequency *f*_ar_, conductance *G*, and the quality factor *Q*_r_ of the SAW resonator. The formulas for calculating these parameters are as follows:
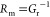
2
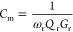
3
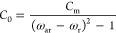
4
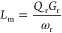
5where *G*_r_ is the peak value of the conductance, ω_r_ = 2π*f* is the angular frequency of the SAW resonator, and ω_ar_ = 2π*f*_ar_ is the angular frequency at antiresonance. The quality factor *Q*_r_ at resonance of SAW resonator is determined as:^37,38^
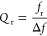
6where Δ*f* is the bandwidth at half of the peak conductance.

The equivalent impedance of the SAW can be obtained from:^38^
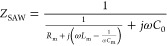
7

At the resonant frequency, the equivalent circuit of the SAW device can be simplified by eliminating nondominant components. The interaction between the motional inductance and capacitance is dominant, and the motional resistance *R*_m_ primarily influences the power loss. The equivalent circuit of the SAW device is shown in Figure 2b with its equivalent impedance is expressed as:^38^
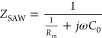
8

### 2.4 Wireless Powered Defogging and Deicing Tests

For the wired defogging and deicing tests, a 9.88 MHz SAW device was utilized to compare it with the wireless powered system. The transmitter and receiver coils (NUCurrent Company model no. 1461798011) had a diameter of 25.02 mm, a thickness of 0.68 mm, and an inductance of 5.00 μH. To match the resonant frequency of the WPT system with that of the SAW device, it is necessary to allow the WPT system to produce minimum reactive power at this frequency. In this system, the primary circuit refers to the transmitter coil and its associated components, while the secondary circuit consists of the receiver coil and the SAW device. However, in the circuit analysis, we did not consider the small reactive power contribution from the SAW device in its resonant frequency and mainly focused on the reactive power and behaviors of the WPT system. To achieve the optimal resonance, the inductance (*L*) and capacitance (*C*) of both the primary and secondary circuits were designed by analyzing the mathematical model of the WPT system. The equation for the imaginary part of the primary and secondary impedances is as follows:^39^
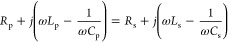
9where ω = 2π*f*, *f* is the resonant frequency of the WPT system designed according to the SAW device. *L*_p_ and *C*_p_ represent the inductance and capacitance values on the primary side of the WPT system, while *L*_s_ and *C*_s_ correspond to the inductance and capacitance values on the secondary side, respectively. *R*_p_ and *R*_s_ denote the impedance values on the primary and secondary sides, respectively. To maximize the transmission efficiency of the WPT system, it is necessary to make the impedance imaginary part of the primary and secondary sides of the WPT system zero:^39^
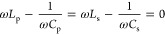
10

The values of *L*_p_, *L*_s_, *C*_p_, and *C*_s_ can be decided by referring to the following equation with the inductance value to be 5 μH, as per the manufacturer.^39^
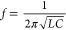
11

In this system design, the compensation structure of the WPT system is a series-series (SS) symmetrical compensation, i.e., *L*_p_ = *L*_s_, *C*_p_ = *C*_s._ Based on the intrinsic resonant frequency of the SAW device, we designed the resonant frequency of the WPT system to be the same as the resonant frequency of the SAW device. In this WPT system, the SAW device is the main load. A set of LCs form a resonant circuit, so the formula for LC and *f* refer to the primary- or secondary-side resonant circuit eq. In this WPT system, a symmetrical SS compensation topology was used, so the circuits on the primary and secondary sides should be symmetrical. Under the operating conditions of this resonant frequency, the reflective power of the whole system showed the minimum value, so that the working efficiency of the system reached the maximum value. Based on the above conditions, the capacitance value was estimated to be 51.9 pF using eq 11, where *f* = 9.88 MHz, *L*_p_ = *L*_s_ = 5 μH.

The distance between the coils in the WPT and the corresponding coupling coefficients is inversely related. Whereas the coupling coefficient *k* is related to the mutual inductance *M* by the following equation:^39^
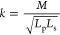
12

The output power of WPT system *P*_out_ can be obtained from:^39^
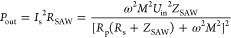
13

In the case of misalignment or distance variation, the key parameter affected is the mutual inductance *M* between the transmitting and receiving coils, which subsequently influences the coupling coefficient *k*.^40^ Essentially, the coupling coefficient determines the system’s efficiency. Since the analysis in this paper is based on a design where the coupling coefficient is a critical parameter, it remains applicable even in the presence of misalignment. Consequently, the experiments presented in this paper primarily investigate the effect of distance variation on system efficiency, examining how efficiency changes under different coupling coefficients and demonstrating the effectiveness of the WPT in powering the SAW device.

A network analyzer was used to evaluate the system’s energy transfer distance *d*, transfer efficiency, and the coupling coefficient *k* at various distances. Based on these assessments, during both the defogging and deicing experiments, the distances between the transmitter and receiver coils were varied from 0.5 to 2.0 cm to find the optimum power transfer efficiency. The RF powers applied for both the defogging and deicing functions for this wireless power transfer system were ranged from 0.24 to 12.10 W, and the same signal generator, RF amplifier and RF power meter were utilized via the transmitter and receiver coils. The amplified signal was applied to the SAW device, and output power from the receiver coil was measured using the RF power meter. The impedance values of the coils and SAW device were measured using the vector network analyzer. During the actuation process, the IR camera was utilized to monitor the temperature changes at various RF powers to study the acousto-thermal effects. The wireless power transfer efficiency was calculated using the following equation:
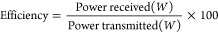
14

## 3 Results and Discussion

### 3.1 Characterization of SAW Devices

**Figure 3:**
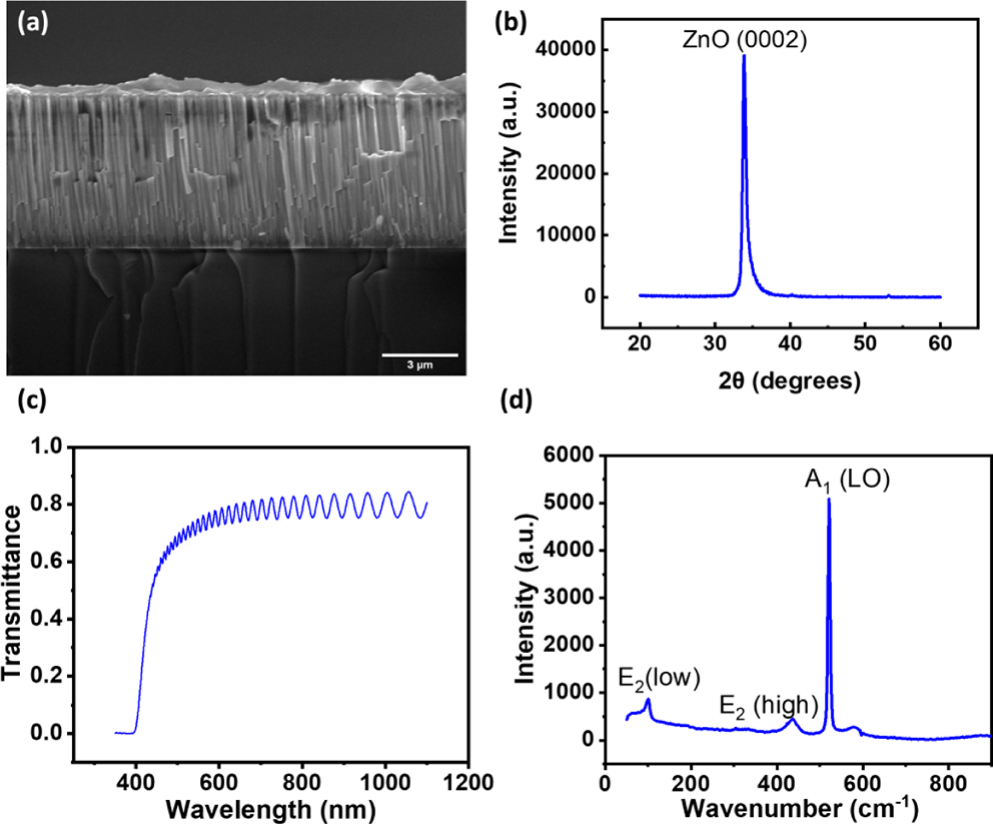
(a) Cross-sectional morphology of ZnO thin film on glass; (b) XRD diffractogram of ZnO thin film on glass; (c) UV–vis transmission spectra of ZnO thin film on glass; (d) Raman spectrum of ZnO thin film on glass.

Figure 3a presents the cross-sectional microstructure of the ZnO thin film deposited on the glass substrate, revealing a vertical columnar structure with a film thickness of approximately 4.5 μm. Figure 3b shows an XRD diffractogram of the ZnO thin film on the glass substrate. A strong and sharp peak was observed at 33.90°, which corresponds to the (0002) plane of the ZnO thin film, showing the *c*-axis preferential growth of the wurtzite ZnO film. Figure 3c shows the transmission spectrum from the UV–vis spectrophotometer of the ZnO thin film on a glass. The optical transmittance value was observed to be greater than 70% within the measured wavelength from 350 to 1100 nm. Figure 3d shows the vibration modes of the ZnO thin film from the Raman spectrum of the ZnO thin film. The peak at approximately 100 cm^–1^ is assigned to the nonpolar modes of *E*_2_ (low). The other two peaks which appear at 435 cm^–1^ and 521 cm^–1^ are corresponding to the *E*_2_ (high) and *A*_1_ (LO) vibration modes of ZnO, respectively. All the above characterization results show the high-quality crystalline structures of the ZnO film on glass substrate.

**Figure 4:**
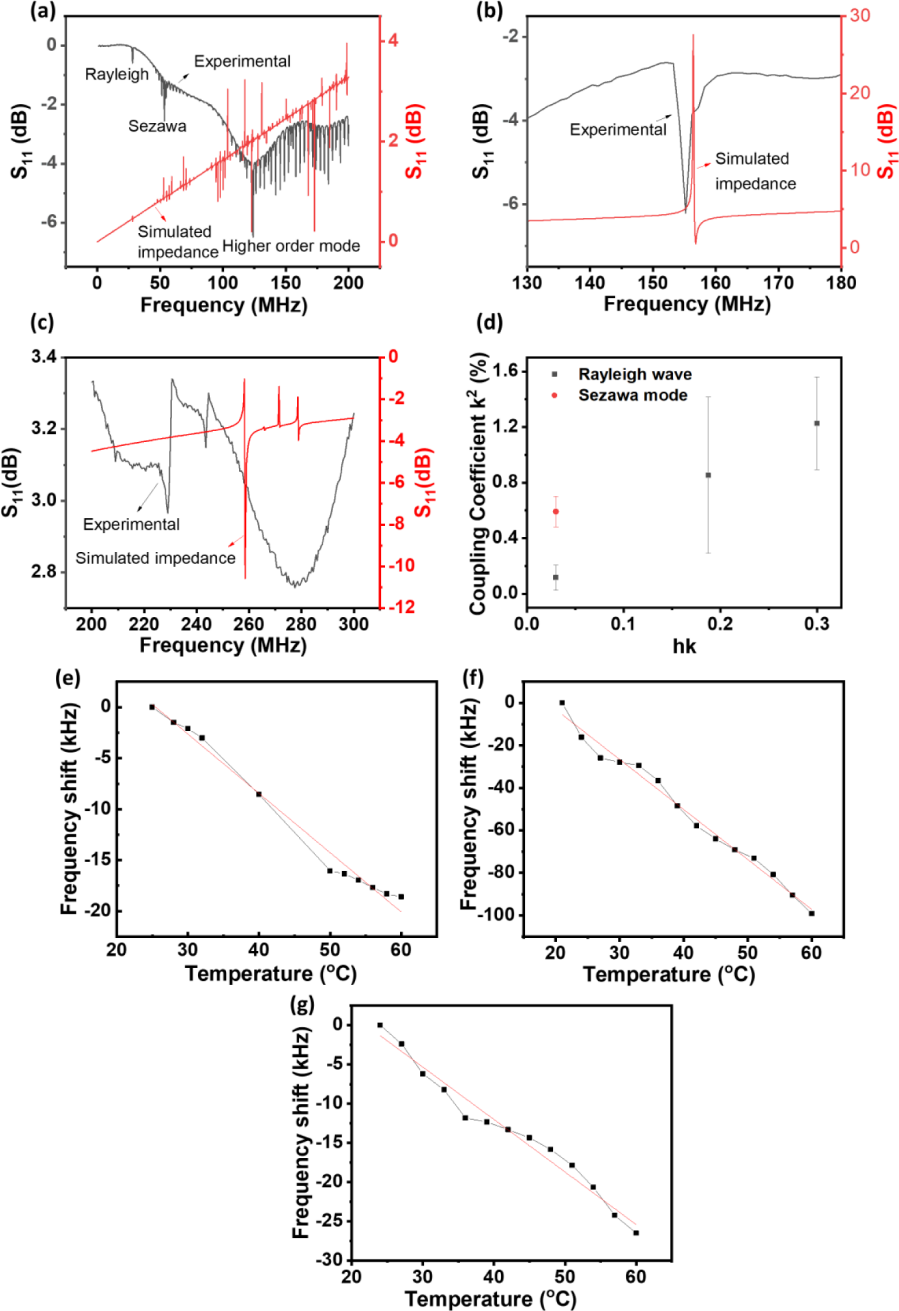
(a–c) S11 spectra of ZnO/glass SAW devices with wavelengths of 100, 16, and 10 μm, respectively; (d) coupling coefficients of ZnO/glass SAW devices; (e–g) TCF values of ZnO/glass SAW devices with wavelengths of 100, 16, and 10 μm, respectively.

Figure 4a–c show examples of the obtained reflection spectra (*S*_11_) of the ZnO/glass SAW device with wavelengths of 100, 16, and 10 μm, respectively. The resonant frequencies of the Rayleigh waves were found to be 28.19, 162.58, and 244.21 MHz, respectively, for the different wavelengths of 100, 16 and 10 μm (corresponding to the velocities of 2819.00, 2601.28, and 2442.1 m/s). For the 100 μm wavelength ZnO/glass SAW device, the Sezawa mode appears at 51.53 MHz and the multiple peaks centered around 126.28 MHz are assigned to the higher-order (harmonic) modes.

Figure 4d shows the obtained average values of the electromechanical coupling coefficient *k*^*2*^ for the Rayleigh waves of the ZnO/glass SAW devices with different wavelengths. The *k*^*2*^ values are changed from 0.12%, 0.85%, to 1.23% with the increase of the normalized thickness. These values are comparable to those published in literature,^34,41,42^ According to a publication by Zhou et al.,^42^ when the wavelength of the SAW devices was increased from 16 to 32 μm, the *k*^*2*^ values of the Rayleigh wave SAW devices was reduced from 1.8% to 0.5%. The energy was largely distributed into the nonpiezoelectric glass as the wavelength was increased, which eventually caused a decrease in the *k*^*2*^ values.^41,42^ The *k*^*2*^ value for the Sezawa mode is 0.59% at a normalized thickness of 0.03, which is similar to those reported in the literature (e.g., 0.45 to 0.50%).^43^ As the Sezawa mode’s frequency is significantly greater, the wave energy is largely contained within the interface and the ZnO thin film, increasing the coupling coefficient in the process.^44−46^ It was also reported that as the wavelength increases, the *k*^*2*^ values of the Sezawa wave mode were decreased.^42^

Figure 4e–g show the frequency shifts versus temperature for the Rayleigh wave mode of ZnO/glass SAW devices, from which the data of TCF were obtained. Based on the graphs, the TCF values were determined to be 44.33, 51.12, and 27.33 ppm/°C for the SAW devices with wavelengths of 100, 16, and 10 μm, respectively. The TCF values of the SAW devices are decreased as the normalized thickness of the piezoelectric ZnO thin film is increased as the acoustic waves propagate more in such layer at lower wavelengths.^47^ ZnO has a negative TCF value, whereas glass normally has a positive TCF value. As the normalized thickness increases, the TCF values get closer to zero, which is excellent for sensing applications as the temperature influence will be minimized significantly. Our obtained values are comparable to those reported in the literature for ZnO/glass SAW devices.^34,47,48^ To be compared with, those TCF values for ZnO/Si and 128 °C Y-cut LiNbO_3_ SAW devices are ranged from −18 ppm/°C to −75 ppm/°C.^34^

### 3.2 Defogging and Deicing Performance of Thin Film SAW Devices with and without WPT System Wireless Power Transfer Efficiency and Acousto-Thermal Effect

**Figure 5:**
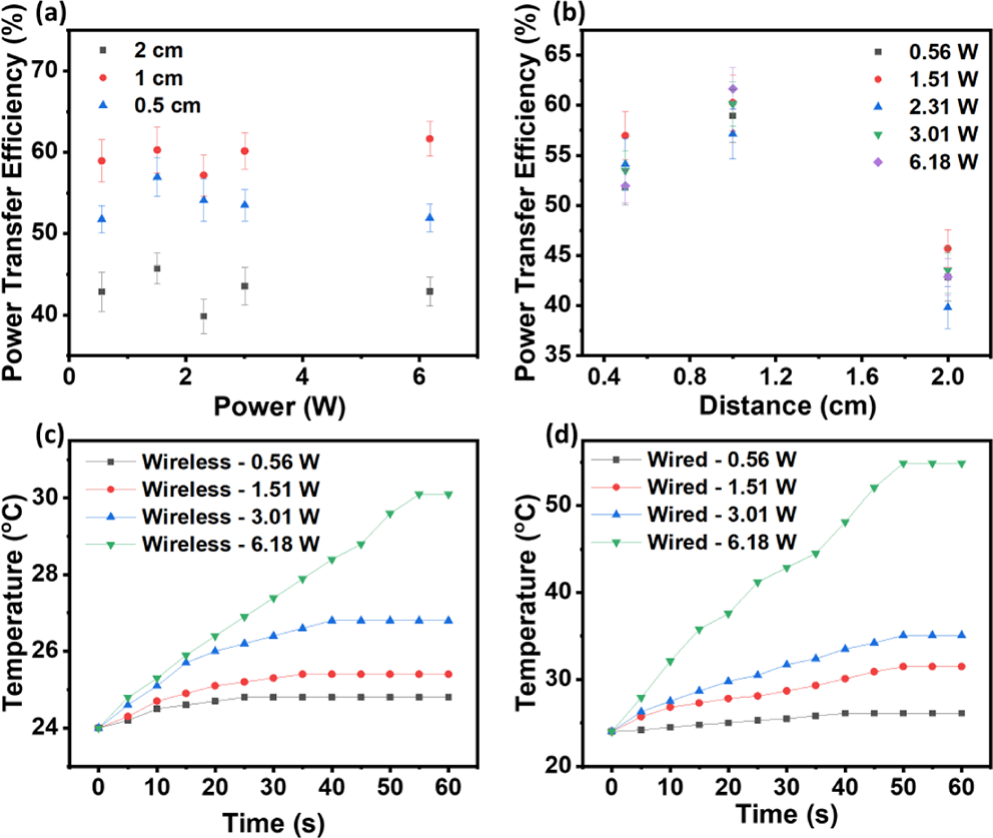
(a) Power transfer efficiency (%) as a function of RF power at different distances of the coils; (b) efficiency vs distance with error bars. IR camera results for (c) wired system at various RF powers and (d) wireless system at various RF powers.

Figure 5a shows the transmission efficiency of the WPT system, which was used to power a SAW device with the different distances between two coils (from 0.5 to 2.0 cm). The transmission efficiency is highest at a distance of 1.0 cm and decreases when the distance is either increased to 2.0 cm or lowered to a distance of 0.5 cm. This is mainly because the transmission efficiency at the critical coupling distance (which is ∼1.0 cm in this study) is the highest in the WPT system. As this distance increases, the coupling coefficient decreases, so the efficiency decreases. When the distance is less than the critical coupling distance, the system is in the overcoupling region, and there is a more pronounced frequency splitting phenomenon as presented in Figure 5a. This was generated as the decreasing distance increased the coupling coefficient, therefore resulting in an overcoupled state. A large coupling coefficient results in splitting of the resonant frequency from one into two different frequencies. The effect of the frequency splitting then reduces the transmission efficiency of the system.

Figure 5b shows the transmission efficiency of a WPT system powering a SAW device at different distances and powers. Variation of power levels was found to change the efficiency, despite being at the same distance. This is due to the problem of impedance matching in the WPT systems; impedance matching between the transmitter and receiver is essential for efficient energy transfer. However, impedance matching is not consistent at all power levels. As the transmitted power varies, the components in the system exhibit different impedance characteristics, leading to changes in the matching conditions and thus affecting the transmission efficiency.

The temperature readings obtained from the IR camera were observed to be lower for the wireless systems compared to those of the wired systems. Figure 5c,d show the IR camera results for both the wired and wireless systems, respectively. The temperature increments for the wired system are much larger than those of the wireless system. This can be explained by the wired system transmitting the RF power directly to the SAW device, prompting the acousto-thermal effect upon SAW agitation. Also, the connection through silver paint for the cable and device often resulted in significant local heating effects for the SAW devices. As for the wireless power system, several factors come into play, including the distance between the two coils, the alignment of coils, and the impedance condition of the load. A single factor or a combination of these factors can have a great effect on the acousto-thermal effect induced by SAW during SAW agitation. All these dramatically reduce the local heating effects and minimize the thermal generation in the wireless powered system.

#### 3.2.1 Defogging and Deicing Using Thin Film SAW Systems with and without Wireless Power System

**Figure 6:**
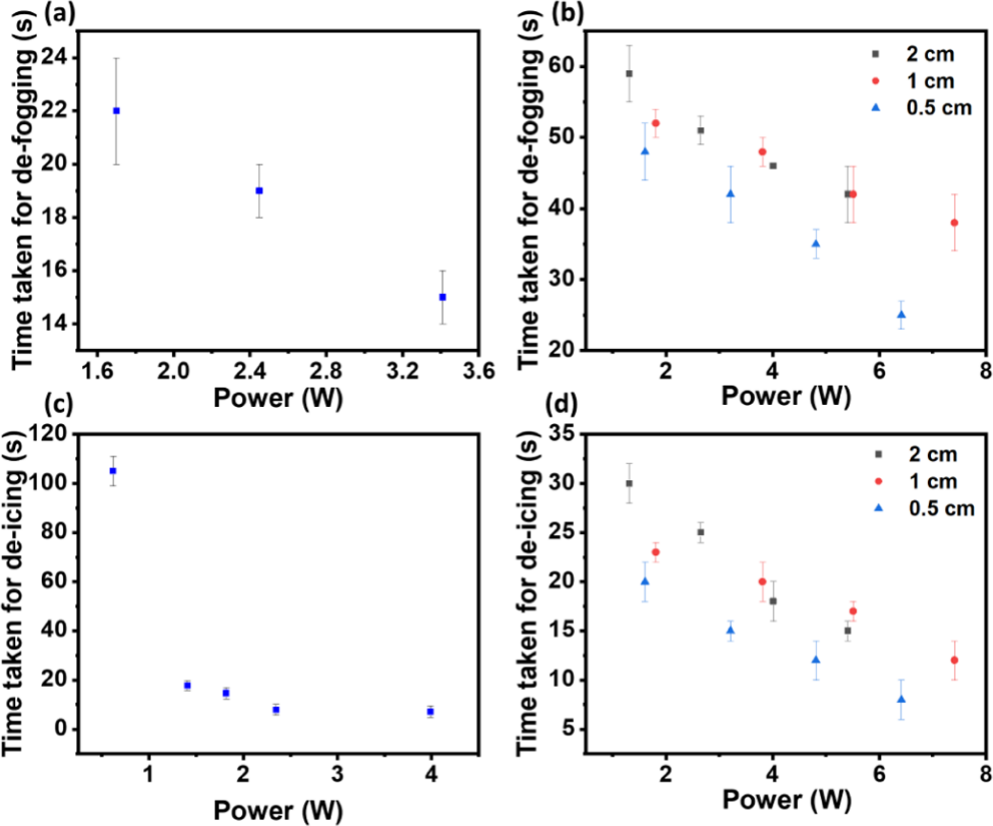
Defogging for (a) wired system at various RF powers and (b) wireless system at various RF powers and distances between two coils. Deicing for (c) wired systemand (d) wireless system at various RF powers and distances between two coils.

Figure 6a,b show the times taken for defogging using the 9.88 MHz SAW device for both wired and wireless SAW systems, respectively. Figure 6a shows that the time taken for defogging is decreased with increasing RF powers with the standard wired system. The observation can be attributed to the mechanical vibrations caused by SAW agitations inducing internal acoustic streaming in the condensates, coupled with a strong localized acousto-thermal effect when SAW RF power is applied to the ZnO/glass SAW device.^23,49^ As the waves propagated across the surface, they effectively drove away or evaporated the condensates, resulting in a defogging action.

Figure 6b shows that the wireless system exhibits a trend similar to that of the wired system, where the time required for defogging was reduced as the RF powers were increased. The distance between the two coils was changed from 0.5 to 2.0 cm, and the obtained results are presented in Figure 6b, which clearly demonstrate that the shorter coil distances lead to reduced defogging times as RF power increases. However, it is worth noting that the wireless system’s defogging process takes much longer time than the wired system, necessitating higher power levels due to losses in the system. This slight increase in the power level is required due to the reduced efficiency of the power transfer occurring through variations in the coil distance and coupling coefficient.

Figure 6c,d show the times taken for rime ice deicing using the 9.88 MHz SAW device for both the wired SAW system and the wireless power system. Similar to the trends observed in Figure 6a,c shows that the time taken for rime ice deicing is decreased with increasing RF powers with the wired system. This can be explained by the mechanical vibrations from the SAW acoustic energy, which causes significant changes at the interfaces of the rime ice clusters with the substrate upon SAW agitations, including microstructures and morphology of the porous rime ice. In addition to the mechanical vibrations caused by the applied RF power, interfacial localized heating (i.e., acoustic thermal effect) was generated on the ZnO thin film or at the interfaces due to SAW energy dissipation.^23^ This would eventually cause shrinkage, melting, or elimination of the porous rime ice. As time was prolonged, the porous rime ice was reduced and eliminated, completing the deicing process due to SAW agitations and the acousto-thermal action.

Figure 6d shows the results obtained with the wireless power system for the deicing of rime ice. It also shows trends similar to those in Figure 6b where the time taken for deicing was reduced as the RF powers were increased. The distance between the two coils was changed from 0.5 to 2.0 cm. Results show clearly that with the decrease of distances between the two coils, less time was required to fulfill the deicing action with increasing applied RF powers. Power transfer efficiency was observed to be the optimized potion at ∼ 1.0 cm, but both the defogging and deicing results were obtained at ∼ 0.5 cm. This observation could be due to the acoustic heating induced during SAW agitations, where the combined effects of the WPT system and SAW allow the defogging and deicing process to occur more efficiently. In addition, the frequency splitting could make the resonant frequency of the WPT system different from the initial conditions. For this study, using the resonant frequency of the SAW device and the input powers, if there is frequency splitting, the system’s impedance should be increased, and thus the efficiency could be reduced.

### 3.3 Ice Sensing/Monitoring Using Thin Film SAW Device

**Figure 7:**
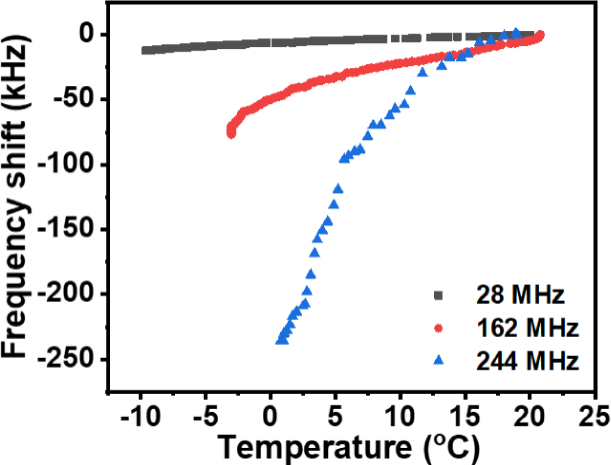
Comparisons of frequency shifts for three different frequencies ZnO/glass SAW devices without exposure to humid air, only cooling down of cold chamber from RT to lower temperature.

Figure 7 illustrates the frequency shifts versus temperature when the ZnO/glass SAW device was subjected to temperature changes in the chamber conditions (without controlling humidity). This is case 1 in the experimental designs (Section 2.2). The resonant frequency was observed to be decreased as the temperature was decreased. In principle, if there was no influence of humidity, the frequency shift should ideally be increased with the decrease in temperature, and the slope should be the same as those from the TCF values. However, all the results obtained here showed otherwise, which suggests that this change was actually caused by the increased relative humidity of the cold chamber. Both the temperature and vapor pressure have significant influences on the water vapor phase transition in a cold environment.^20^ Fogging was created when the saturated air was cooled down and releases its moisture.^20^ Water vapor was directly converted from a gaseous to a solid state when it encountered a cold surface which was less than the freezing temperature of water or the dew-point temperature. The moisture in the chamber was condensed on the SAW devices as the temperature was reduced, which caused a mass loading effect on the SAW device, resulting in an increased reduction in the frequency.

The data of TCF was obtained from Figure 7, which shows the frequency shifts of the SAW devices when they were cooled down from room temperature to lower temperatures. The obtained TCF values were determined to be 14.23, 17.29, and 57.40 ppm/°C for the devices with frequencies of 28, 162, and 244 μm, respectively. As compared to the TCF values obtained at room and higher temperature ranges, the TCF values obtained in case 1 for both samples of 28 and 162 MHz were observed to be slightly lower, whereas the TCF value obtained for the sample of 244 MHz was observed to be slightly higher. The higher values for the device with higher frequencies can be explained by the fact that wave propagation was mostly within the ZnO film and not in the glass substrate. However, all the obtained TCF values show small TCF values.

**Figure 8:**
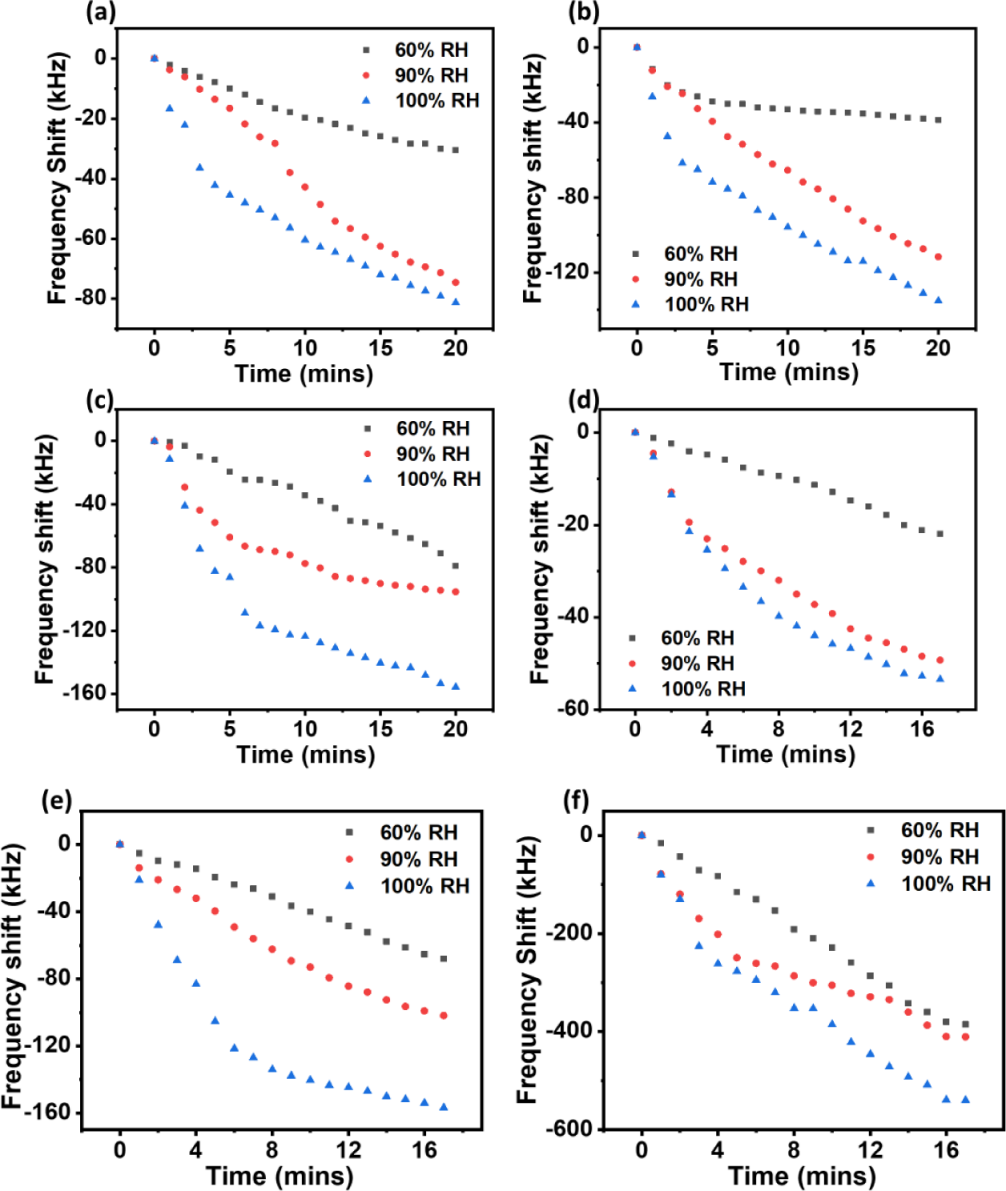
Frequency shifts for the tests without cooling down of cold chamber but with only flowing with humid air for the samples with different frequencies of (a) 28 MHz; (b) 162 MHz; (c) 244 MHz; Frequency shifts by cooling down the chamber first, and then blowing humid air at different intensities for the samples with different frequencies of (d) 28 MHz; (e) 162 MHz; (f) 244 MHz.

Figure 8a–c illustrate the frequency shifts versus time as the cold chamber was exposed to humid air at different relative humidities (RH 60, 90, and 100 %) at room temperature. This is case 2 in the experimental designs. It was observed that the frequency shifts were decreased as the time was increased for all the different humid air intensities. As the humid air intensity was increased, more condensates were generated on the surface of the SAW device, and the mass loading effect induced by the condensates became more prominent by the noticeable decreasing shift in frequency as time progressed. A relatively linear trend for the RH 60% case suggests that humid air exposure induced a consistent formation of droplets on the surface, especially for RH 60%. With the increase of humidity levels from RH 60% to 90%, an increase in the flow rate was observed, which caused more rapid formation of droplets on the device’s surface. As the relative humidity was increased, the chamber reached a saturation point much quicker; therefore, droplets were formed on the device surface quickly. This led to a larger accumulation of droplets at the same duration when the RH level was increased. The sharp decrease in the frequency was caused by increased droplet formation. However, when the formed droplets became coalesced, and puddles of water was formed, a gradual decrease in the frequency was observed as shown in Figure 8a–c.

We hypothesized that there are a lot of small droplets formed at the initial stage, which resulted in rapid decreases in frequency shifts. In the second stage, due to a layer of liquid film formation, a gradual decrease in frequency shifts was found. Overall, the frequency shifts were similar for both two stages; however, their differences differed in the droplet size and the time taken. Additionally, the mass loading effect was increased as the humid air was increased, resulting in a massive shift in the frequency compared with the other relative humidities. In a cold climate, mass loading effects were resulted from the adsorption of molecules, which formed a thin layer such that an estimation of frequency shift could be obtained.^20^ In addition to the mass loading effect, the absorption of water molecules on the surface of the SAW device could also generate electrical loading effect, and it frequently occurs in environments where the saturation and dew-point temperatures of ambient water vapor are not reached.^20^ This electrical loading effect will also change the frequency shift.

Figure 8d–f illustrate the frequency shifts versus time as the cold chamber was first cooled down from room temperature to a lower temperature of −9.0 ± 0.3 °C (cold environment), followed by the exposure to the humid air of different relative humidities (RH 60, 90, and 100%). This is corresponding to the case 3 in the experimental designs. It was observed that the frequency shift was decreased as time was increased, and the frequency shift was reduced as the humid air intensities applied was increased. This observation could be due to the built-up ice layer on the surface of the SAW device since the resonant frequencies were decreased significantly and continuously with the increase in time. Frequencies were obviously shifted with the icing process, as ice formation on the surface changed the mass loading and conductivity of the SAW devices. Moreover, a linear line was observed for the case of RH 60% condition, whereas a slight curved shape was observed for those of RH 90% and RH 100% condition. Those curved lines shown in Figure 8d–f illustrate that for 90 and 100 RH% humid air intensities, the frequency shifts were caused by significant droplet condensation on the device’s surface before freezing at the much higher humidity levels. The rapid decrease of the frequency occurred as the droplets were condensed, and a gradual decrease was observed when they began to freeze. At RH 60%, there were more droplets on the surface, and therefore, they were frozen much quicker, which resulted in the significant changes in frequency shifts. The thickness of rime ice was increased with humidity levels as the volume of water droplets condensed and subsequently those nucleated and frozen on the surface were increased while the time remained constantly. In all the cases during the humid air exposure at different intensities, the mass loading effect was dominant during the ice formation process, and this further resulted in the reduction of the frequency as time progressed.

The difference between the results obtained from cases 1 and 3 was that a thin layer of fog was formed in case 1, whereas an ice layer was formed in case 3. The mass loading effects caused by the fog layer formation and frozen ice layer on the surface of the SAW device were dramatically different.^20^ For the fog layer formation, it was assumed that the moisture layer was too thin to cause any significant frequency shift.^20^ However, when there was a significance rime ice formation, this caused large increases of acoustic impedance values and thus more significant changes in frequency shifts.^49^

**Figure 9:**
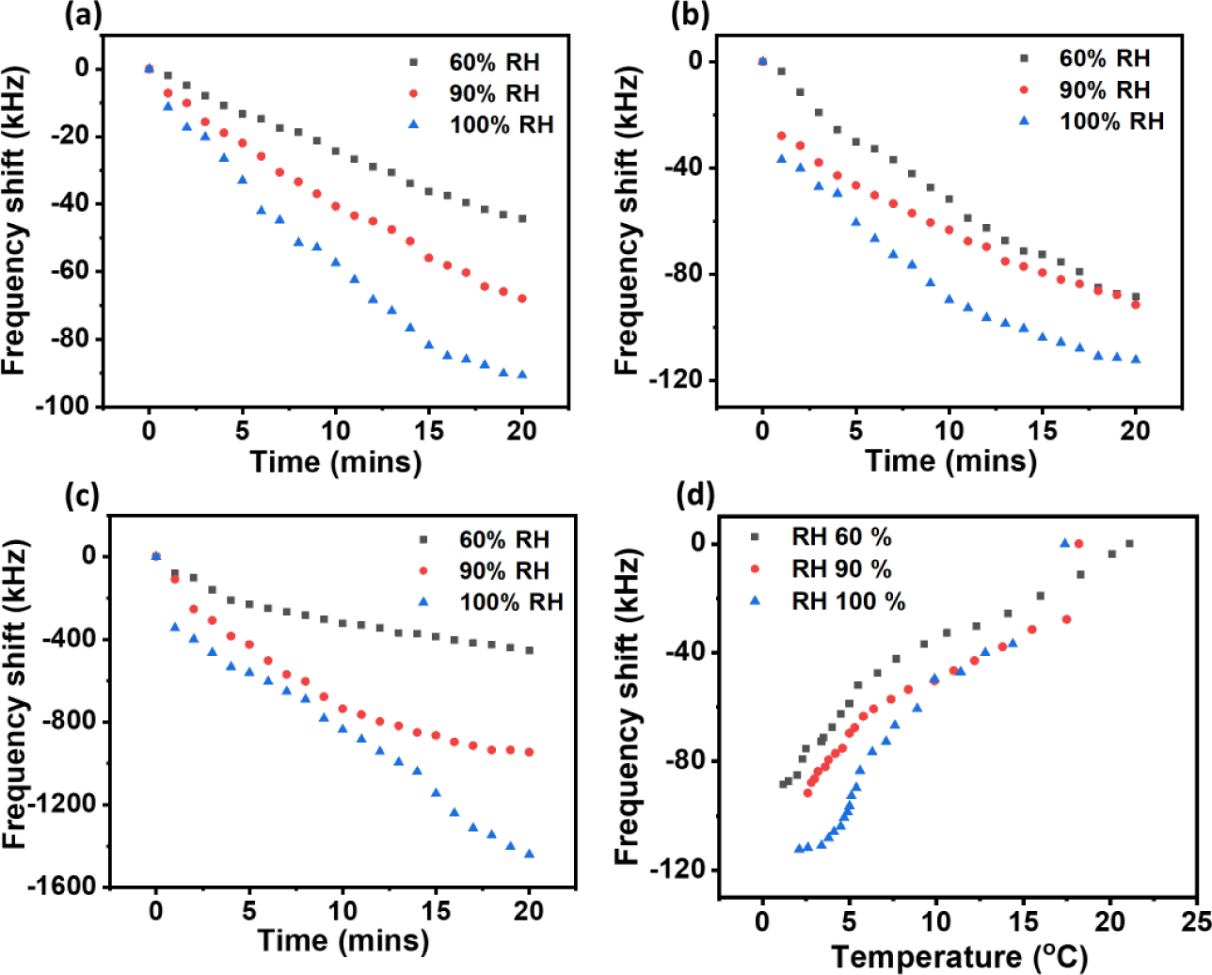
Frequency changes of the SAW devices when cooling down of the cold chamber and blowing humid air at different intensities concurrently for SAW devices with frequencies of (a) 28 MHz; (b) 162 MHz; and (c) 244 MHz. (d) Frequency shift vs temperature for the device with a frequency of 162 MHz.

Figure 9a–c illustrate the frequency shifts vs time as the cold chamber was subjected to cooling down and simultaneously exposed to humid air at different relative humidities (RH 60, 90, and 100%). This corresponds to case 4 in the experimental designs. When the exposure of vapor to the different relative humidities was increased, it was found that the frequency shifts were increased. The frequency shifts were observed to be lower at RH 60% when compared to other intensities, possibly because the cooling process was more dominant compared with humid air exposure. The frequency shifts were found to be largest at the RH 100% relative humidity because humid air exposure was more noticeable than the cooling process in the cold chamber. The shifts appeared to show a change in the system from a temperature-dominant process to a humidity-dominant process. This is possibly due to the relative temperature of the water in the nebulizer being higher than that of the chamber.

Figure 9d presents the frequency shift versus temperature for the 162 MHz. It can be observed that as the temperature decreased, the frequency shift was decreased. The initial decrease in the frequency shift at RH 60% was of a linear line, implying that the change in frequency was mainly due to temperature effects. As for both RH 90% and RH 100%, the decrease in frequency shifts was observed to be greater as temperature decreases. As the temperature was continuously decreased with the humid airflow into the cold chamber, a combination of temperature, humidity, and mass-loading effects caused the significant frequency shifts. After this period, further decreased frequency shifts was due to the ice layer formed on the surface of the SAW device.

## 4 Conclusions

This work utilized transparent thin film SAW technology on glass substrates for monitoring, defrosting, and deicing, with and without using WPT systems. In a cold chamber, the resonant frequency shifts of ZnO/glass SAW devices under various changing conditions were investigated and their correlations with temperature, moisture/humidity, and frequency functions were determined. The results indicate decreased frequency shifts as the ZnO/glass SAW devices were exposed to different environmental factors. The ZnO/glass SAW system was then employed for deicing and defogging applications, and the effects of acousto-thermal and RF powers on the deicing efficiency were assessed. The findings demonstrated that increasing RF power reduced the time required for effective deicing and defogging. Additionally, we observed that higher RF power levels resulted in increased temperatures. Furthermore, we compared the wireless power transfer system with conventional wired SAW systems regarding their heating effects during deicing. It was shown that the acousto-thermal effects of the wireless power transfer system were lesser than those of the wired system, even if the wireless power transfer system required a little more time (about 40–60% more) to complete the deicing and defogging process. These findings highlight the potential of wireless SAW technology for efficient and optically compatible SAW systems despite operational speed trade-offs.
